# Influence of the Area per Player in Non-Professional Soccer Players: A Pilot Study Focused on Positional Roles

**DOI:** 10.3390/ijerph18189833

**Published:** 2021-09-18

**Authors:** Annamaria Mancini, Daniela Vitucci, Pasquale Meo, Adriano Capobianco, Domenico Martone, Francesca Cozzolino, Pasqualina Buono, Esther Imperlini, Stefania Orrù

**Affiliations:** 1 Dipartimento di Scienze Motorie e del Benessere, Università degli Studi di Napoli Parthenope, 80133 Naples, Italy; annamaria.mancini@uniparthenope.it (A.M.); pako_meo88@hotmail.it (P.M.); adriano.capobianco@studenti.uniparthenope.it (A.C.); domarto@gmail.com (D.M.); francesca.cozzolino@collaboratore.uniparthenope.it (F.C.); pasqualina.buono@uniparthenope.it (P.B.); 2 CEINGE-Biotecnologie Avanzate, 80133 Naples, Italy; vitucci@ceinge.unina.it; 3 IRCCS SDN, 80133 Naples, Italy; esther.imperlini@unina.it

**Keywords:** small-sided games, area per player, metabolic power, soccer positional roles

## Abstract

This study analyses the influence of different area per player (A_P_; 75, 98 and 131 m^2^) on the average metabolic power (MP) and other soccer-related performance variables in relation to the positional roles. We recruited 19 non-professional male soccer players (25.2 ± 6.3 y; 23.7 ± 2.3 kg/m^2^; 16.4 ± 6.3 y soccer experience) to play three different small-sided games (SSGs): SSG1 (5 vs. 5; 30 × 30 m; 5 min), SSG2 (5 vs. 5; 35 × 45 m; 5 min) and SSG3 (7 vs. 7; 35 × 45 m; 8 min). Specific playing rules were applied. GPS-assessed soccer-related variables were: average MP (AMP), distance covered in 1 min (DIS); % time spent at high speed (v > 16 km/h; % hst) or MP (>20 W/kg; % hmpt); % distance covered at high positive/negative speed (2 < v < 4 m/s^2^, % ACC; −6 < v < −2 m/s^2^, % DEC); and number of actions at high MP (hmpa). All recorded variables differed when each SSG was compared to the others (*p* < 0.05), but for hmpa for attackers. Most performance variables were positively associated with increasing A_P_ (*p* < 0.05), but for % ACC and % DEC, and differed among positional roles within the same SSG (*p* < 0.05). Here the general applicability of SSGs, regardless the physical/technical skills of the group of players, to enhance performance is confirmed; furthermore, quantitative advices on AMP and other performance variables are provided to achieve significant improvements in all soccer players of the team.

## 1. Introduction

Small-sided games (SSGs) are a form of conditioning in football [1,2,3] and represent an effective alternative to traditional interval training in order to improve players’ endurance with a concomitant physical, cognitive and technical/tactical development [1,2,4,5,6,7]. SSGs are now very popular because they are suitable not only for elite football players, but also for healthy and unhealthy amateurs; they enhance several components of the health-related fitness: in fact, recreational football, carried out as SSGs, has a high aerobic component (80–85% maximum heart rate, HRmax), provides a high impact on muscles and bones, and improves maximum aerobic power blood pressure and body composition [8,9,10,11,12,13].

Many SSG parameters can be modulated to achieve a specific technical/tactical improvement by changing the pitch dimension, the number of players, the game duration, the work/rest ratios, by selecting specific playing rules, by adding goalkeepers, and by using coach encouragement [2,14,15,16]. The prompt availability of balls, when out of play, and coach encouragement are the most applied rules in many experimental settings as both of them are able to increase the intensity of the game [14]. As for the other modifiable parameters, it is challenging to define their effects on specific technical/tactical variables when they are evaluated individually. For example, in small pitch dimensions many features of the match play are well simulated, but high-intensity and repeated-sprint demands are not easily reproduced [6,17]; larger pitch dimensions overestimate high-speed running in respect to match play [18]. In addition, the SSG experimental settings from current research are extremely variable and output data appear fragmented in age groups (preferentially adolescent, or elite or older soccer players), performance measures and pitch configuration. [2,14,19,20,21].

As a matter of fact, an effective SSG relies not only on the application of the single parameters quoted above but also on derived factors, such as the relative pitch area, also called area per player (A_P_). SSGs characterized by match-derived A_P_ (~320 m^2^) show an increase in inter-team and intra-team distances, and allow to better simulate the tactical demands of the official match, above all with a larger number of players [22,23].

Nevertheless, many SSG formats are characterized by lower A_P_, usually less than 200 m^2^ [2,24,25,26]; such SSGs show an higher player density, while reducing both the space for running and the time available to make technical/tactical decisions [23].

Randers and colleagues showed that keeping constant A_P_, while changing the number of players, had a negligible effect on the total covered distance and on the total distance covered per minute [21]; moreover, the same authors found that increasing A_P_, while decreasing the number of players, determined higher recorded values in both variables [27,28]. When the effect of six different A_P_ on physiological response and technical skills in two young soccer groups (under 12, U-12, and under 14, U-14) was investigated, exercise intensity, assessed as % HRmax, increased going from 40 m^2^ to 150 m^2^ [25].

Lately, a different indicator, the metabolic power (MP), was proposed to estimate the energy cost of high-demanding locomotor activities in team sports. The rationale behind the MP approach is based on the energetic equivalence between an accelerated running on a flat terrain and an uphill running at constant speed [29,30,31]. Despite this model of analysis was debated [32,33,34], some improvements were proposed [35] and it is now widely used to classify locomotion intensity in team sports by means of global positioning system (GPS) devices [36,37].

In the last decade technology has gained an instrumental role in quantifying the demand in training and competitions [38]. GPS devices represent an effective support to the analysis of SSG variables and are now common tools to assess players’ performance in team sports [39]. GPS receivers are considered reliable and have been validated in several team sport motion activities [21,30,31,40,41]. GPS technology is able to evaluate the total distance and/or the time spent in a specific soccer-related kinematical variable [30]; moreover, it allows the MP and the estimated energy cost during acceleration and/or deceleration activities to be quantified, in order to better calculate, through a mathematical approach, the metabolic and mechanical load of soccer players [42].

In this scenario, the present pilot study investigated the influence of different A_P_ on the average MP (AMP) and other soccer-related performance variables in non-professional male soccer players in correlation with their positional role. A scheme of SSGs, similar to that proposed by Gaudino and coll. [31] on 26 elite soccer players in the English Premiere League, was adopted. Accordingly, the A_P_ selected were: 75, 98 and 131 m^2^. Soccer-related performance variables included: distance, distance covered at high positive/negative speed, time spent at high speed/MP, number of actions at high MP. Positional roles included attackers, midfielders and defenders. The aims of the study were: (i) to acquire scientific evidence regarding the effects of specific SSGs on players with different skills and (ii) to provide quantitative information on the minimal A_P_ increment that each positional role required to achieve significant improvements on AMP and on the other soccer-related performance variables.

## 2. Materials and Methods

### 2.1. Participants

Data were collected over a 10-week period from 19 non-professional male soccer players (age: 25.2 ± 6.3 years; height: 1.76 ± 0.06 m; weight: 74.2 ± 7.2 kg; BMI: 23.7 ± 2.3 kg/m^2^; soccer experience: 16.4 ± 6.3 years) participating to the regional championships in Italy during the in-season competition period. Recruited subjects trained regularly 3 times a week (90 min/session) and played a competitive match once a week.

Inclusion criteria for participants were: a soccer experience longer than 10 years, no partial/chronic injuries, normal vision (or corrected to normal), no history of neuropsychological impairment. All 19 soccer players matched the inclusion criteria and none were excluded.

Each player was instructed on the scope of the study, including its potential benefits and risks, and signed an informed consent (approval code by Ethical Committee of University of Naples “Federico II”: n. 376/19). The study was conducted according with the ethical standards of the Declaration of Helsinki of 2013.

### 2.2. Study Design

In order to acquire scientific evidence regarding the effects of specific SSGs on players with different skills, the adopted small sided game (SSG) scheme resembled one previously proposed by Gaudino et al. [31] on 26 elite soccer players in the English Premiere League. Three different SSGs were considered: SSG1 (5 vs. 5; 30 × 30 m) and SSG2 (5 vs. 5; 35 × 45 m) drills lasted 5 min, whereas in SSG3 (7 vs. 7; 35 × 45 m) soccer players were monitored for 8 min (Table 1). The explored AP were: 75, 98 and 131 m^2^.

Players were assigned to one of the following positional roles: attacker, midfielder and defender. To achieve a high work-rate, each game was supervised by encouraging coaches and the ball was promptly returned when out of play [43]; other specific playing rules were applied, such as 3 m-goals and ball possession limited to three consecutive touches [44,45]; moreover, in this study defenders could not go beyond the midfield line, attackers could not retreat behind the midfield line. Substitutions were not allowed; scores were not evaluated. The external load was assessed by global positioning system (GPS) devices.

SSGs were planned weekly during the standard training and were conducted on an outdoor synthetic-grass soccer pitch at the same time of day (between 6 and 8 p.m.) to limit the effects of circadian variations on heart rate measurements [46].

The training session included a 20-min warm-up, a 60-min section based on technical/tactical development, and a final 10-min cool down.

Before playing a specific SSG, the 20-min warm-up consisted of activities without the ball, such as low-intensity running, striding, skipping and stretching.

### 2.3. Physical Variables, Instruments, Softwares

AMP was assessed using wearable GPS-devices (Q-Starz, Taipei, Taiwan) sampling at 10 Hz. This GPS unit has excellent static and dynamic validity in a variety of settings [47,48].

GPS devices were located in a tiny pocket at the upper back of each player (above shoulder blades) and activated at least 10 min before the start of the data collection to allow the connection to satellites [31,49]. Besides AMP (W/kg), other soccer-related variables were considered as listed in Table 2.

Only GPS-based horizontal data were considered and speed was determined as horizontal position differentiation over time. The analysis was performed by the “LagalaColli_Bridge4” software (now available as GPSLAGALACOLLI at Spinitalia S.r.l., Pomezia, Italy) [40,50,51] in accordance with the manufacturer guidelines.

Due to the different duration of SSG3 compared to SSG1 and SSG2 (Table 1), physical performance parameters (Table 2) were considered as percentage (% hst, % hmpt, % ACC, % DEC) or are normalized to 1 min (AMP, DIS, hmpa) to allow comparison among the formats.

The Di Prampero equation [42], successively modified by Osgnach [29], was used to estimate the energy cost and MP:

EC = (155.4·ES5 − 30.4·ES4 − 43.3·ES3 + 46.3·ES2 + 19.5·ES + 3.6)·EM·KT(1)

where EC = energy cost of accelerated running on grass (J/Kg·m); ES = equivalent slope (= tan(90 − arcan·g/a_f_)); g = Earth’s acceleration gravity; a_f_ = forward acceleration; EM = equivalent of body mass = [(a_f_^2^/g^2^) + 1]^0.5^**;** KT = 1.29 (constant). Hence,

MP(W/Kg) = EC·v(2)

where v = velocity (m/s).

All parameters were calculated according to Osgnach [29] considering that the time spent at MP > 20 W/kg is energetically equivalent to an oxygen uptake of 57 mL/kg·min (above resting), whereas the time spent at v > 16 km/h is energetically equivalent to an accelerated/decelerated run at 20 W/kg [29].

### 2.4. Statistical Analysis

The data were presented as means and standard deviations (mean ± SD). Significant differences among training game formats (SSG1, SSG2, SSG3) in relation to the positional roles (or vice versa) were determined by one-way ANOVA applied to each of the dependent variables (AMP, % hst, % hmpt, % ACC, % DEC, DIS, hmpa). Whenever a significant difference was found, Fisher’s post hoc test was used.

The effect size with 95% confidence intervals (CI) was calculated by means of unbiased Hedge’s g equation, suggested to correct bias associated with small size samples [52], and interpreted as follows: <0.20: trivial; 0.20–0.59: small; 0.60–1.19: moderate; 1.20–1.99: large; ≥2.00: very large [53,54].

Two-way mixed ANOVA [within-subjects factor: SSG (SSG1, SSG2, SSG3); between-subjects factor: time-related soccer variables (% hst and % hmpt)] was performed; in the presence of positive overall effect of time-related soccer variables and/or of SSG, Fisher’s post hoc test was carried out.

All the statistical analysis were performed using Statview (Version 5 0.1) for Windows, with significance being set at *p* < 0.05.

Pearson’s correlation was used to determine the correlation coefficient between the measured variables in each positional role and the different A_P_ (75, 98 and 131 m^2^) of the SSGs.

## 3. Results

The specific playing rules adopted in this pilot study affected the performance of the players in relation to their positional roles; nevertheless, interesting findings emerged on average MP and the other GPS-recorded variables by analysing soccer-specific performance within the different A_P_, obtained by changing the number of players or the pitch dimensions (Table 1).

Table 3 shows the mean ± SD for each analyzed soccer-related performance variable according to players’ positional role within a specific SSG; statistical analysis was performed comparing data among SSGs for the same positional role (rows) and among roles within the same SSG (columns).

Most recorded variables differed when each SSG was compared to the others (rows; *p* < 0.05) or when attacker’s, midfielder’s and defender’s performances were compared within the same SSG (columns; *p* < 0.05).

Table 4 shows the one-way ANOVA analysis related to the pairwise comparison of any analyzed variable among SSGs for the same positional role and among roles within the same SSG.

The correlation between the measured soccer-related variables in each positional role and the different A_P_ (75, 98 and 131 m^2^) of the explored SSGs was evaluated, by means of Pearson’s correlation coefficient, as shown in Table 5.

### 3.1. Defenders

As expected, for this positional role the lowest soccer-related values were recorded compared to attackers and midfielders, in almost all SSGs (Table 3).

Comparing defenders’ performance among SSGs showed that the highest values were found in SSG2 and they always displayed a large/very large correlation from those recorded in SSG1 (Table 4), except for % distance covered at high positive/negative speed (% ACC and % DEC); in addition, the covered distance per minute (DIS) was different from the correspondent measure in SSG1 (103.5 ± 8.0 m vs. 91.8 ± 5.3 m, *p* < 0.05; ES: 1.37). Conversely, % ACC and % DEC showed the lowest values in SSG3 compared to SSG1 and SSG2 with a very large effect size, while no significant differences were detected between SSG1 and SSG2 (Table 4).

When defenders’ performances were then correlated to the three proposed formats (SSG1, SSG2 and SSG3) by Pearson’s correlation analysis, the MP-related variables (AMP, % hmpt and hmpa), the distance covered in a minute (DIS) and the % time spent at high speed (% hst) were found to be positively associated to increasing A_P_ (Table 5).

### 3.2. Attackers

Although they moved into a similar smaller area, attackers showed sometimes significantly different GPS-recorded values than defenders (Table 3 and Table 4). Analyzing the attackers’ performance among SSGs, we found that AMP, DIS, and the % time spent at high speed or at high MP (% hst, % hmpt) in SSG2 were higher from those recorded in SSG1 with a very large effect size. A significant difference was observed in the same variables, except for the % time spent at high speed, between SSG3 and SSG1 with a large effect size. In addition, % time spent at high metabolic power by attackers in SSG2 was moderately higher than the corresponding measure in SSG3 (15.7 ± 1.9 vs. 13.5 ± 1.9, *p* < 0.05, ES: 1.06). As for % distance covered at high positive/negative speed (% ACC and % DEC), the lowest detected values were recorded in SSG3, differing from those in SSG1 and SSG2 with a very large correlation (Table 3 and Table 4).

For this role, the recorded values for the average MP, DIS, and the % time spent at high speed/MP (% hst and % hmpt) were found to be positively associated with the increasing A_P_ by Pearson’s correlation analysis (Table 5).

### 3.3. Midfielders

The only restrained playing rule for midfielders was “ball possession limited to three consecutive touches”; hence, unlike defenders and attackers, they could play in the whole available area.

Comparing midfielders’ performance among SSGs showed that most variables were higher in SSG2 in respect to the other two formats. All the analyzed variables in SSG2, except for DIS, were significantly different from SSG3 with a moderate-to-very-large correlation (Table 3 and Table 4), whereas the average MP, DIS, and the % time spent at high speed/MP (% hst and % hmpt) differed between SSG2 and SSG1 with a very large effect size. Finally, only midfielders’ performance in % time spent at high speed largely differed between SSG3 and SSG1 (3.5 + 1.2 vs. 1.7 + 0.6, *p* < 0.01, ES: 1.59).

Due to their specific playing condition, the midfielders showed the highest GPS-recorded values for the MP-related and DIS variables compared to the other two positional roles in SSG1 and SSG2 with large/very large effect size; a significant difference between midfielders and defenders or attackers was also observed for AMP and DIS in SSG3 with an effect size from moderate to very large (Table 3 and Table 4). Furthermore, % time spent at high speed (% hst) outclassed defenders’ correspondent parameter in all the formats with a large/very large effect size, while a large correlation was found for % time spent/the number of action at high MP (% hmpt and hmpa) between midfielders and defenders in SSG3. The recorded values for % distance covered at high positive/negative speed (% ACC and % DEC) did not differ in comparison to the other positional roles in all formats except for a large correlation observed in the % DEC in SSG2 (Table 3 and Table 4).

Pearson’s correlation analysis, comparing the three proposed formats (SSG1, SSG2 and SSG3) to midfielders’ performances, showed a positive correlation between AMP, DIS, % time spent at high speed or at high MP (% hst and % hmpt) and increasing A_P_ (Table 5).

### 3.4. High Speed versus High Metabolic Power (MP)

**Figure 1:**
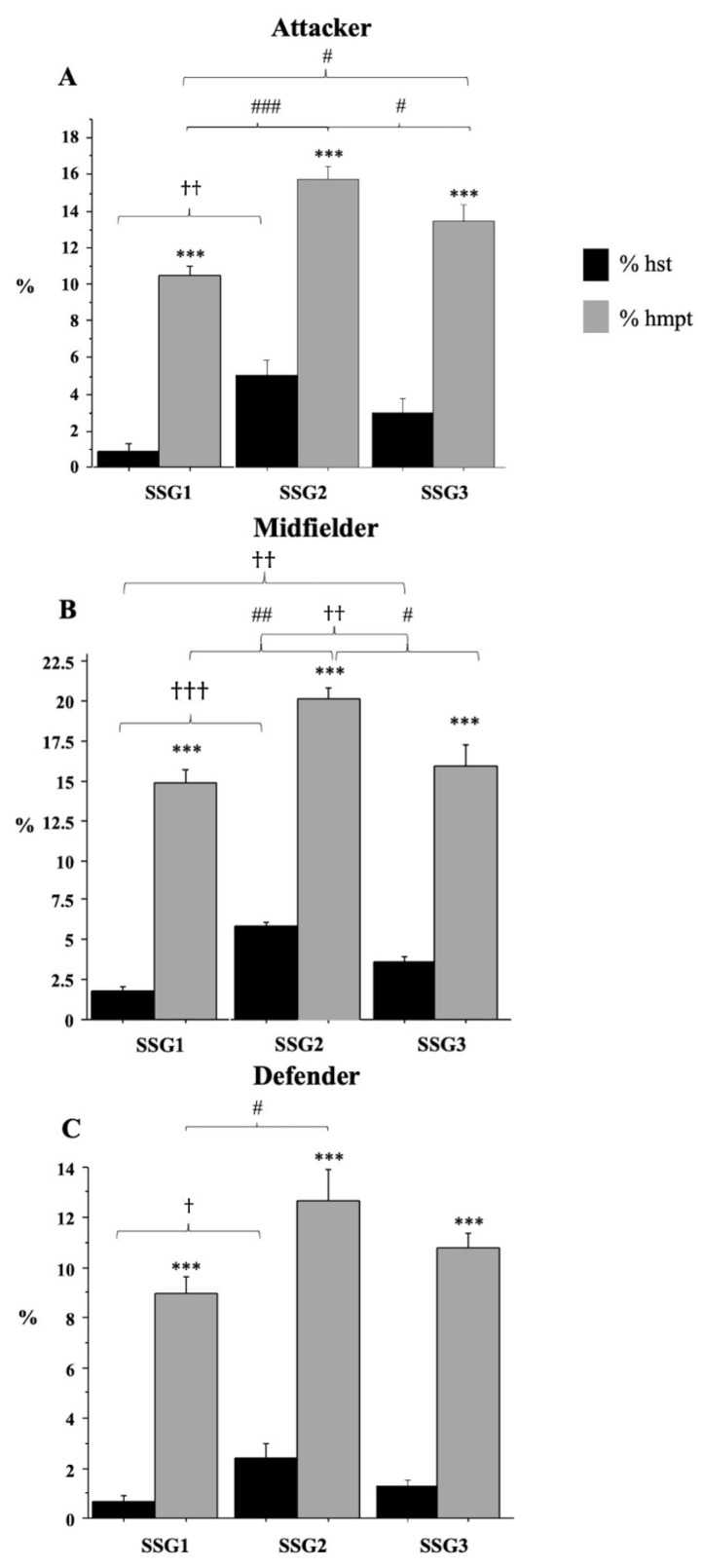
Percentage of total time spent at high speed (% hst; v > 16 km/h) and at high metabolic power (% hmpt; MP > 20 W) recorded in SSG1, SSG2 and SSG3 for attacker (**A**), midfielder (**B**) and defender (**C**). Values are expressed as mean ± SD. * Significant difference between % hst and % hmpt in the same SSG (*** *p* < 0,001); † Significant difference between % hst in different SSGs († *p* < 0.05; †† *p* < 0.01; ††† *p* < 0,001); # Significant difference between % hmpt in different SSGs (# *p* < 0.05; ## *p* < 0.01; ### *p* < 0,001).

We then compared the % time spent at high speed (% hst) vs. high MP (% hmpt), observing that % hmpt was greater than % hst across all SSGs and positional roles (*p* < 0.0001; Figure 1). For midfielders, the highest % hst or % hmpt values were recorded in SSG2 and they were statistically different from those in SSG1 and SSG3 (see legend of Figure 1B for details). A similar difference (*p* < 0.05) was found for attackers, but for the comparison of % hst between SSG2 and SSG3 (Figure 1A). As for defenders, the only significant difference was found for % hst and % hmpt values between SSG1 and SSG2 (Figure 1C).

## 4. Discussion

The purpose of this pilot study was to examine the influence of different A_P_ on the average metabolic power (AMP) and on other soccer-specific performance variables (DIS, % hst, % hmpt, hmpa, % ACC, % DEC). To this aim non-professional male soccer players were recruited and their performance was analyzed in relation with positional roles (attacker, midfielder and defender). Specific playing rules were adopted and applied in all SSGs; in particular, defenders could not go beyond the midfield line, and attackers could not retreat behind the midfield line. Such rules affected the performance of the players within their positional roles; nevertheless, interesting findings emerged when defenders’ vs. attackers’ or midfielders’ vs. attackers’ performances were compared. In particular, we found not only significant differences in the variables recorded between defenders and attackers, but also there were examples where no differences were found between midfielders and attackers.

Within a specific SSG, midfielders’ performance, assessed as MP-related variables (AMP, % hmpt, hmpa) and as distance covered in a minute (DIS), outclassed the other two roles in SSG1 and SSG2 with a moderate to very large correlation. A similar correlation was observed for AMP and DIS in SSG3. In several cases, also attackers performed better than defenders in SSG1 (AMP, hmpa) with a large correlation and in SSG2 (AMP, % hst, % hmpt, DIS) with a moderate/large correlation, but not in SSG3. Moreover, we did not find any differences between attackers and midfielders in SSG1 (% hst, % ACC, % DEC), in SSG2 (% hst, % ACC) and in SSG3 (% hst, % hmpt, hmpa, % ACC, % DEC). These observations suggest that, despite the playing rules applied in this study, the roles are distinguishable as well as the players’ profiles. In fact, as expected, regardless of the same conditions (A_P_, rules), attackers drove the game more than defenders resulting in better AMP, or longer distances, or longer times spent at high speed or high MP. For the same reasons, attackers’ performance was indistinguishable from midfielders, despite the different playing spatial constrains, in relation with time spent or covered distance at high speed. These findings are in agreement with previous studies carried out also on elite players [16,55,56,57,58], confirming that the positional role specifies the performance profile.

The SSG1 and SSG3 formats resembled SSG 5 vs. 5 and SSG 7 vs. 7, respectively, reported by Gaudino et al. [30,31] on soccer players competing in the English Premiere League, where both the number of players and the pitch dimension changed. Therefore, an intermediate format (SSG2) was added to better understand the effects of such parameters on soccer-related performance variables (Table 1). In fact, moving from SSG1 to SSG2, only the pitch dimension increased; then, moving from SSG2 to SSG3, more players were allowed in the same larger pitch dimension. Such a scheme allowed three different A_P_ to be explored, from 75 to 131 m^2^, where the added format shared a similar A_P_ in respect with SSG 10 vs. 10 from Gaudino et al. [30,31]. Our data confirm that high-intensity demands of soccer training are underestimated by traditional measurements of running speed alone [30,31]. In fact, regarding the ability to assess high intensity demands as function of % hst or % hmpt, all players, regardless of their positional role, spent less time in high speed actions compared to high MP actions, but such a difference decreased with increasing A_P_. This finding corroborates that in smaller SSGs the acceleration/deceleration actions are kept at the expense of high speed actions. Moreover, similarly to Gaudino et al. [30], the lowest % hst and % hmpt values recorded for defenders suggest their playing profile, where the brief explosive actions against opposing attackers are more than hmpa [31,56].

Additionally, despite the different level of recruited players and the different playing rules, the distance covered in 1 min in SSG1 and SSG2 by non-professional players, according to their positional role, was similar or slightly higher compared to Premiere League players, whereas, in SSG3, DIS data were similar or slightly lower [30,31]. Such a finding confirms the general applicability of SSGs, regardless the physical/technical skills of the group of players, to enhance performance [59].

Another goal achieved in this pilot study was the opportunity to quantitatively evaluate which parameters, affecting A_P_, were instrumental to obtaining an improvement in a specific soccer-related variable within each positional role considered.

Most performance parameters within a specific positional role were positively associated to increasing A_P_ according to Pearson’s correlation (Table 5); on the other hand, the % distance covered at high positive/negative speed (% ACC and % DEC) did not show any correlation.

Defenders showed the lowest recorded variables compared to attackers and midfielders in all SSGs. GPS-recorded values relative to MP-related variables (AMP, % hmpt, hmpa) and to % time spent at high speed (% hst) increased going from the smaller to the wider A_P_; but, largely significant improvements were observed only with a 75% increase in A_P_ (from SSG1 to SSG2), and not with a lower one (31% from SSG1 to SSG3, and 34% from SSG3 to SSG2). The distance covered in 1 min (DIS) was instead affected by the pitch dimension thus determining a largely significant difference only between SSG1 (900 m^2^) and the other two formats (1575 m^2^). As for the distance covered at high positive/negative speed (% ACC and % DEC), these variables were affected by the number of players in the format: in fact, the lowest significant values were recorded in SSG3 (7 vs. 7) compared to SSG1 and SSG2 (both 5 vs. 5; *p* < 0.001; ES: > 2.00).

Despite playing in half-field, attackers often performed better than defenders. AMP and DIS values varied significantly in relation with the pitch dimension (SSG1 vs. SSG2 or SSG3) with a large/very large correlation. % Time spent at high MP (% hmpt) values were positively associated to A_P_, indicating that a 30% increase in A_P_ was sufficient to achieve the expected improvement (*p* < 0.05) with a moderate to very large correlation, and that this parameter was sensitive both to increased number of players and to increased pitch dimension; on the other hand, % time spent at high speed (% hst) showed significant improvements only with a 75% increase in A_P_ (SSG1 vs. SSG2) with a very large correlation. Conversely, no correlation was found for hmpa in the different played SSGs. Like defenders, % ACC and % DEC values referring to attackers’ performance were affected by the number of players in the format with a very large effect size.

Unlike defenders and attackers, midfielders’ playing actions did not have spatial limits, hence their performances were always better than the other two roles. Like defenders and attackers, their best performance was recorded in SSG2, the format having the widest A_P_. In particular, AMP and % time spent at high speed or at high MP (% hst and % hmpt) values correlated positively with increasing A_P_ and data from SSG2 differed significantly from those recorded in SSG1 and SSG3 with a moderate to very large correlation. Between similar variables of SSGI and SSG3 there were no significant differences; the only exception was represented by % time spent at high speed (% hst) that was longer in the larger field. The distance covered in a minute in SSG2 by midfielders differed from the corresponding value in SSG1 with a very large correlation (75% increase in A_P_), whereas hmpa values in SSG2 largely differed in respect to SSG3. Also for this positional role, a very large effect size was observed when % ACC and % DEC values were compared between SSGs having a different number of players involved (SSG3 vs. SSG1 or SSG2).

Therefore, it is apparent that, unlike most analyzed variables, % ACC and % DEC are definitely not sensitive to increasing A_P_, but the key determinant to gain an improvement is the number of players in the format, regardless of the players’ positional roles. This evidence is in line with previous reports, where comparing the same number of players in two different pitch areas (60 vs. 80 m^2^) did not show any differences in the distance covered while accelerating or decelerating [28].

The present pilot study has some limitations. The sample size (*n* = 19 participants) cannot be considered representative with regard to the aims of the study. A higher sample size would have improved confidence in the generalizability of the results. Despite this, the effect sizes of the soccer-related variables, evaluated in the pairwise comparison (Table 4), often show a large/very large correlation coupled to significantly differences observed among SSGs/positional roles.

In addition, no internal load measurements (HR or rating of perceived exertion) were performed; however, MP data show a strong relationship with both walking and running activities [36], therefore providing a different tool for this purpose.

## 5. Conclusions

This pilot study quantitatively describes A_P_-affecting determinants able to significantly improve soccer-specific performance variables, demonstrating that different arrays of SSGs are needed to fulfill the demands related to players’ positional role, irrespective of their physical/technical level. Soccer coaches are aware that a single SSG always has high positive effects on health-related fitness components, but it is not always possible to adequately train a soccer-specific variable for all positional roles; here, quantitative advice on AMP and other performance variables is provided to achieve significant improvements in all soccer players of the team.

In summary, to record a significant improvement in the performance of the three MP-related parameters (AMP, % hmpt and hmpa), defenders need a 75% increase of A_P_, whereas for midfielders a 30–40% increment is sufficient; attackers behave similarly to midfielders, except for hmpa: none of the explored SSGs allowed an improvement to be achieved in the number of actions at high MP in a minute for this role. On the other hand, DIS and % hst variables were more affected by the selected playing rules, displaying a similar behavior for attackers and defenders compared with midfielders. In particular, DIS improved significantly for attackers and defenders with a 30–40% increase in A_P_ while midfielders required a 75% increment. Conversely, the percentage of time spent at high speed (% hst) showed an opposite trend (a 30–40% increase in A_P_ for midfielders, a 75% increase in A_P_ for attackers and defenders). For all positional roles, % ACC and % DEC values were sensitive only to the number of players.

## Figures and Tables

**Table 1:** Small-sided game (SSG) features evaluated in this study.

Drill	Format	Duration (min)	Pitch Dimension (m)	Pitch Area (m^2^)	Area per Player (m^2^)
SSG1	5 vs. 5	5	30 × 30	900	75
SSG2	5 vs. 5	5	35 × 45	1575	131
SSG3	7 vs. 7	8	35 × 45	1575	98

**Table 2:** Soccer-related performance variables analyzed in this study.

Variable	Acronym	Unit
Average metabolic power	AMP	W/kg
distance covered in 1 min	DIS	m/min
time spent at high speed (v > 16 km/h) as percentage	% hst	-
time spent at high metabolic power (MP > 20 W/kg) as percentage	% hmpt	-
distance covered at high positive speed (accelerations; 2 < v < 4 m/s^2^) as percentage	% ACC	-
distance covered at high negative speed (decelerations; −6 < v < −2 m/s^2^) as percentage	% DEC	-
number of actions per minute performed at high metabolic power	hmpa	number/min

**Table 3:** Mean values ± SD of the variables recorded in the SSGs considered.

Variable	Position	SSG 1	SSG 2	SSG 3	F-Value	*p*-Value
AMP	Attacker	10.0 ± 0.8 ^b^	12.0 ± 0.9 *^,b^	11.3 ± 0.9 *	F(2, 15) = 7.8	*p* < 0.01
	Midfielder	11.9 ± 0.5 ^a,b^	13.7 ± 0.7 *^,#,a,b^	12.5 ± 1.2 ^a,b^	F(2, 15) = 6.1	*p* < 0.05
	Defender	9.1 ± 0.5	10.7 ± 1.3 *	10.2 ± 0.9	F(2, 16) = 3.9	*p* < 0.05
F-value		F(2, 12) = 25.1	F(2, 17) = 15.1	F(2, 17) = 8.6		
*p*-value		*p* < 0.001	*p* < 0.01	*p* < 0.01		
% hst	Attacker	0.9 ± 0.8	5.0 ± 2.3 *^,b^	2.9 ± 2.1	F(2, 15) = 6.6	*p* < 0.01
	Midfielder	1.7 ± 0.6 ^b^	5.8 ± 0.8 *^,#,b^	3.5 ± 1.2 *^,b^	F(2, 15) = 25.0	*p* < 0.001
	Defender	0.7 ± 0.4	2.4 ± 1.6 *	1.3 ± 0.6	F(2, 16) = 4.0	*p* < 0.05
F-value		F(2, 12) = 3.9	F(2, 17) = 7.3	F(2, 17) = 4.6		
*p*-value		*p* < 0.05	*p* < 0.01	*p* < 0.05		
% hmpt	Attacker	10.5 ± 1.1	15.7 ± 1.9 *^,#,b^	13.5 ± 1.9 *	F(2, 15) = 12.7	*p* < 0.001
	Midfielder	14.9 ± 1.8 ^a,b^	20.1 ± 1.9 *^,#,a,b^	15.9 ± 3.5 ^b^	F(2, 15) = 6.4	*p* < 0.01
	Defender	9.0 ± 1.4	12.7 ± 3.1 *	10.8 ± 1.6	F(2, 16) = 3.9	*p* < 0.05
F-value		F(2, 12) = 21.1	F(2, 17) = 14.9	F(2, 17) = 7.3		
*p*-value		*p* < 0.001	*p* < 0.01	*p* < 0.01		
hmpa	Attacker	3.9 ± 0.7 ^b^	4.7 ± 0.8	4.2 ± 1.0	F(2, 15) = 1.3	*p* > 0.05
	Midfielder	5.0 ± 0.9 ^a,b^	5.7 ± 0.9 ^#,a,b^	4.5 ± 0.5 ^b^	F(2, 15) = 3.8	*p* < 0.05
	Defender	2.8 ± 0.2	3,9 ± 0.9 *	3.5 ± 0.8	F(2, 16) = 3.7	*p* < 0.05
F-value		F(2, 12) = 12.5	F(2, 17) = 6.9	F(2, 17) = 3.3		
*p*-value		*p* < 0.01	*p* < 0,01	*p* > 0.05		
% ACC	Attacker	2.7 ± 0.3 ^#^	2.8 ± 0.5 ^#^	1.4 ± 0.2	F(2, 15) = 22.9	*p* < 0.001
	Midfielder	2.6 ± 0.4 ^#^	2.9 ±0.3 ^#^	1.3 ± 0.2	F(2, 15) = 48.7	*p* < 0.001
	Defender	2.4 ± 0.3 ^#^	2.7 ± 0.4 ^#^	1.5 ± 0.2	F(2, 16) = 24.3	*p* < 0.001
F-value		F(2, 12) = 0.8	F(2, 17) = 0.4	F(2, 17) = 1.7		
*p*-value		*p* > 0.05	*p* > 0.05	*p* > 0.05		
% DEC	Attacker	3.5 ± 0.4 ^#^	3.3 ± 0.4 ^#^	1.9 ± 0.2	F(2, 15) = 37.1	*p* < 0.001
	Midfielder	3.9 ± 0.8 ^#^	3.9 ± 0.5 ^#,a,b^	2.1 ± 0.2	F(2, 15) = 23.8	*p* < 0.001
	Defender	3.2 ± 0.3 ^#^	3.2 ± 2.4 ^#^	1.9 ± 0.2	F(2, 16) = 59.4	*p* < 0.001
F-value		F(2, 12) = 1.9	F(2, 17) = 6.0	F(2, 17) = 2.1		
*p*-value		*p* > 0.05	*p <* 0.01	*p* > 0.05		
DIS	Attacker	100.2 ± 8.7	121.1 ± 7.6 *^,b^	114.3 ± 9.2 *	F(2, 15) = 9.0	*p <* 0.01
	Midfielder	117.7 ± 5.3 ^a,b^	137.3 ± 7.0 *^,a,b^	128.1 ± 11.5 ^a,b^	F(2, 15) = 6.8	*p <* 0.01
	Defender	91.8 ± 5.3	106.5 ± 12.1 *	103.5 ± 8.0 *	F(2, 16) = 3.9	*p <* 0.05
F-value		F(2, 12) = 19.7	F(2, 17) = 17.6	F(2, 17) = 11.3		
*p*-value		*p* < 0.01	*p* < 0.001	*p* < 0.01		

AMP = average metabolic power; % hst = % high speed time (v > 16 km/h); % hmpt = % high metabolic power time (MP > 20 W); % ACC = % distance at high positive speed (accelerations; 2 < v < 4 m/s^2^); % DEC = % distance at high negative speed (decelerations; −6 < v < −2 m/s^2^); DIS = total distance; hmpa = number of high metabolic power actions (MP > 20 W).* Significantly different (*p* < 0.05) from SSG1; ^#^ Significantly different (*p* < 0.05) from SSG3; ^a^ Significantly different (*p* < 0.05) from attacker; ^b^ Significantly different (*p* < 0.05) from defender.

**Table 4:** Pairwise comparison of recorded variables among roles within the same SSG and among SSGs within the same positional role and. *p* values, effect sizes and confidence intervals are reported.

Pairwise Comparison among Roles within the Same SSG					
Variable	Drill	Position	*p*-Value	ES	CI
AMP	SSG1	Midfielder vs. Attacker	**<0.001**	2.44	0.99/4.42
		Midfielder vs. Defender	**<0.001**	4.87	2.72/8.05
		Attacker vs. Defender	**0.037**	1.24	−0.01/2.75
	SSG2	Midfielder vs. Attacker	**0.007**	1.99	0.78/3.51
		Midfielder vs. Defender	**<0.001**	2.69	1.33/4.45
		Attacker vs. Defender	**0.022**	1.14	0.07/2.35
	SSG3	Midfielder vs. Attacker	**0.047**	1.05	−0.04/2.31
		Midfielder vs. Defender	**<0.001**	1.98	0.80/3.42
		Attacker vs. Defender	0.084	1.06	−0.02/2.33
% hst	SSG1	Midfielder vs. Attacker	0.062	1.02	−0.21/2.46
		Midfielder vs. Defender	**0.021**	1.87	0.53/3.60
		Attacker vs. Defender	0.567	0.33	−0.88/1.62
	SSG2	Midfielder vs. Attacker	0.419	0.42	−0.65/1.55
		Midfielder vs. Defender	**0.002**	2.43	1.13/4.09
		Attacker vs. Defender	**0.011**	1.25	0.17/2.49
	SSG3	Midfielder vs. Attacker	0.451	0.33	−0.74/1.45
		Midfielder vs. Defender	**0.009**	2.17	0.96/3.67
		Attacker vs. Defender	0.054	0.99	−0.09/2.24
% hmpt	SSG1	Midfielder vs. Attacker	**<0.001**	2.58	1.09/4.61
		Midfielder vs. Defender	**<0.001**	3.24	1.58/5.58
		Attacker vs. Defender	0.127	1.10	−0.13/2.57
	SSG2	Midfielder vs. Attacker	**0.004**	2.11	0.88/3.67
		Midfielder vs. Defender	**<0.001**	2.57	1.32/4.42
		Attacker vs. Defender	**0.034**	1.07	0.01/2.27
	SSG3	Midfielder vs. Attacker	0.106	0.77	−0.31/1.96
		Midfielder vs. Defender	**0.001**	1.77	0.63/3.15
		Attacker vs. Defender	0.067	1.42	0.29/2.76
hmpa	SSG1	Midfielder vs. Attacker	**0.028**	1.18	−0.05/2.67
		Midfielder vs. Defender	**<0.001**	2.96	1.39/5.19
		Attacker vs. Defender	**0.028**	1.85	0.51/3.57
	SSG2	Midfielder vs. Attacker	**0.045**	1.16	0.06/2.45
		Midfielder vs. Defender	**0.002**	1.81	0.62/3.27
		Attacker vs. Defender	0.128	0.82	−0.22/1.97
	SSG3	Midfielder vs. Attacker	0.552	0.31	−0.76/1.43
		Midfielder vs. Defender	**0.024**	1.46	0.36/2.74
		Attacker vs. Defender	0.093	0.82	−0.27/2.01
% ACC	SSG1	Midfielder vs. Attacker	0.604	0.28	−0.93/1.56
		Midfielder vs. Defender	0.491	0.39	−0.82/1.69
		Attacker vs. Defender	0.237	0.79	−0.41/2.18
	SSG2	Midfielder vs. Attacker	0.506	0.38	−0.71/1.48
		Midfielder vs. Defender	0.371	0,53	−0.54/1.68
		Attacker vs. Defender	0.806	3.19	1.69/4.90
	SSG3	Midfielder vs. Attacker	0.347	0.46	−0.61/1.59
		Midfielder vs. Defender	0.081	1.01	−0.02/2.22
		Attacker vs. Defender	0.424	0.39	−0.65/1.56
% DEC	SSG1	Midfielder vs. Attacker	0.231	0.62	−0.59/1.96
		Midfielder vs. Defender	0.075	0.99	−0.23/2.43
		Attacker vs. Defender	0.504	0.62	−0.58/1.97
	SSG2	Midfielder vs. Attacker	**0.014**	1.23	0.12/2.53
		Midfielder vs. Defender	**0.004**	1.70	0.54/3.14
		Attacker vs. Defender	0.588	5.54	3.36/8.09
	SSG3	Midfielder vs. Attacker	0.153	0.80	−0.28/2.00
		Midfielder vs. Defender	0.067	0.90	−0.14/2.07
		Attacker vs. Defender	0.705	0.22	−0.86/1.32
DIS	SSG1	Midfielder vs. Attacker	**0.001**	2.18	0.79/4.05
		Midfielder vs. Defender	**<0.001**	4.41	2.41/7.36
		Attacker vs. Defender	0.069	1.05	−0.18/2.50
	SSG2	Midfielder vs. Attacker	**0.006**	2.05	0.82/3.59
		Midfielder vs. Defender	**<0.001**	2.82	1.43/4.62
		Attacker vs. Defender	**0.009**	0.66	−0.39/1.76
	SSG3	Midfielder vs. Attacker	**0.020**	1.22	0.11/2.52
		Midfielder vs. Defender	**<0.001**	2.32	1.08/3.87
		Attacker vs. Defender	0.062	1.17	0.06/2.45
**Pairwise Comparison among SSGs within the Same Positional Role**					
**Variable**	**Position**	**Drills**	***p*-Value**	**ES**	**CI**
AMP	Attacker	SSG1 vs. SSG2	**0.001**	2.16	0.85/3.8
		SSG1 vs. SSG3	**0.031**	1.33	0.12/2.79
		SSG3 vs. SSG2	0.141	0.79	−0.29/1.99
	Midfielder	SSG1 vs. SSG2	**0.004**	2.74	1.26/4.71
		SSG1 vs. SSG3	0.277	0.55	−0.58/1.77
		SSG3 vs. SSG2	**0.025**	1.16	0.06/2.44
	Defender	SSG1 vs. SSG2	**0.014**	1.42	0.23/2.84
		SSG1 vs. SSG3	**0.063**	1.39	0.21/2.80
		SSG3 vs. SSG2	0.415	0.38	−0.66/1.46
% hst	Attacker	SSG1 vs. SSG2	**0.003**	2.06	0.78/3.69
		SSG1 vs. SSG3	0.108	1.08	−0.09/2.47
		SSG3 vs. SSG2	0.072	0.88	−0.20/2.09
	Midfielder	SSG1 vs. SSG2	**<0.001**	5.15	2.98/8.24
		SSG1 vs. SSG3	**0.006**	1.59	0.39/3.07
		SSG3 vs. SSG2	**<0.001**	2.03	0.80/3.55
	Defender	SSG1 vs. SSG2	**0.015**	1.24	0.08/2.61
		SSG1 vs. SSG3	0.341	1.08	−0.07/2.40
		SSG3 vs. SSG2	0.075	0.84	−0.20/1.99
% hmpt	Attacker	SSG1 vs. SSG2	**<0.001**	2.90	1.44/4.85
		SSG1 vs. SSG3	**0.014**	1.67	0.41/3.23
		SSG3 vs. SSG2	**0.039**	1.06	−0.04/2.31
	Midfielder	SSG1 vs. SSG2	**0.005**	2.52	1.10/4.41
		SSG1 vs. SSG3	0.517	0.32	−0.80/1.51
		SSG3 vs. SSG2	**0.012**	1.37	0.24/2.69
	Defender	SSG1 vs. SSG2	**0.014**	1.31	0.14/2.71
		SSG1 vs. SSG3	0.205	1.06	−0.09/2.38
		SSG3 vs. SSG2	0.133	0.72	−0.32/1.85
hmpa	Attacker	SSG1 vs. SSG2	0.126	0.98	−0.16/2.28
		SSG1 vs. SSG3	0.475	0.38	−0.78/1.62
		SSG3 vs. SSG2	0.378	0.45	−0.62/1.59
	Midfielder	SSG1 vs. SSG2	0.148	0.72	−0.44/2.02
		SSG1 vs. SSG3	0.318	0.61	−0.52/1.83
		SSG3 vs. SSG2	**0.015**	1.54	0.40/2.92
	Defender	SSG1 vs. SSG2	**0.015**	1.50	0.31/2.95
		SSG1 vs. SSG3	0.117	1.08	−0.06/2.43
		SSG3 vs. SSG2	0.267	0.50	−0.53/1.60
% ACC	Attacker	SSG1 vs. SSG2	0.824	0.11	−1.03/1.26
		SSG1 vs. SSG3	**<0.001**	3.99	2.18/6.51
		SSG3 vs. SSG2	**<0.001**	3.05	1.62/4.95
	Midfielder	SSG1 vs. SSG2	**0.097**	0.84	−0.32/2.17
		SSG1 vs. SSG3	**<0.001**	3.79	2.06/6.03
		SSG3 vs. SSG2	**<0.001**	5.82	3.62/8.90
	Defender	SSG1 vs. SSG2	0.172	0.64	−0.48/1.88
		SSG1 vs. SSG3	**<0.001**	3.52	1.83/5.81
		SSG3 vs. SSG2	**<0.001**	3.15	1.69/5.09
% DEC	Attacker	SSG1 vs. SSG2	0.496	0.33	−0.81/1.50
		SSG1 vs. SSG3	**<0.001**	4.80	2.81/7.75
		SSG3 vs. SSG2	**<0.001**	4.11	2.40/6.43
	Midfielder	SSG1 vs. SSG2	**0.097**	0.01	−1.17/1.21
		SSG1 vs. SSG3	**<0.001**	2.98	1.49/4.94
		SSG3 vs. SSG2	**<0.001**	4.36	2.77/7.17
	Defender	SSG1 vs. SSG2	0.940	0.04	−1.11/1.19
		SSG1 vs. SSG3	**<0.001**	4.55	2.60/7.40
		SSG3 vs. SSG2	**<0.001**	5.07	3.10/7.83
DIS	Attacker	SSG1 vs. SSG2	**<0.001**	2.39	1.05/4.15
		SSG1 vs. SSG3	**0.014**	1.44	0.21/0.92
		SSG3 vs. SSG2	0.167	0.76	−0.32/1.95
	Midfielder	SSG1 vs. SSG2	**0.002**	2.83	1.34/4.85
		SSG1 vs. SSG3	**0.060**	1.02	−0.13/2.32
		SSG3 vs. SSG2	**0.081**	0.87	−0.21/2.09
	Defender	SSG1 vs. SSG2	**0.015**	1.37	0.19/2.77
		SSG1 vs. SSG3	**0.047**	1.52	0.28/3.03
		SSG3 vs. SSG2	0.547	0.22	−0.86/1.33

Bold font was used in order to highlight significant *p* value.

**Table 5:** Pearson’s correlation between the measured variables and the different A_P_ of the SSGs.

		Attackers	Midfielders	Defenders
AMP	*r*	0.691	0.668	0.533
	*p*	**0.001**	**0.003**	**0.018**
% hst	*r*	0.681	0.877	0.575
	*p*	**0.002**	**<0.001**	**0.010**
% hmpt	*r*	0.779	0.659	0.570
	*p*	**<0.001**	**0.003**	**0.011**
hmpa	*r*	0.388	0.397	0.544
	*p*	0.111	0.103	**0.016**
% ACC	*r*	0.200	0.317	0.350
	*p*	0.425	0.200	0.141
% DEC	*r*	0.108	0.150	0.189
	*p*	0.670	0.552	0.439
DIS	*r*	0.706	0.682	0.513
	*p*	**0.001**	**0.002**	**0.025**

Bold font was used in order to highlight significant *p* value. *r* Pearson’s correlation coefficient; *p p*-value.
